# The effect of preoperative synbiotic treatment to prevent surgical-site infection in hepatic resection

**DOI:** 10.3164/jcbn.19-46

**Published:** 2019-10-30

**Authors:** Hiroya Iida, Masaya Sasaki, Hiromitsu Maehira, Haruki Mori, Daiki Yasukawa, Katsushi Takebayashi, Mika Kurihara, Shigeki Bamba, Masaji Tani

**Affiliations:** 1 Department of Surgery, Shiga University of Medical Science, Seta Tsukinowa-cho, Otsu, Shiga 520-2192, Japan; 2 Division of Clinical Nutrition, Shiga University of Medical Science, Seta Tsukinowa-cho, Otsu, Shiga 520-2192, Japan

**Keywords:** synbiotic treatment, synbiotics, surgical-site infection, hepatectomy, hepatic resection

## Abstract

We aimed to clarify the influence of preoperative synbiotic therapy on surgical-site infections (SSIs) after hepatic resection. Between January 2011 and December 2017, 284 patients who underwent hepatic resection without biliary tract reconstruction and resection of other organs were included. We prospectively administered *Clostridium butyricum* and partially hydrolyzed guar gum before hepatic resection between April 2016 and December 2017 (synbiotic group). One-hundred-fifteen patients of the synbiotic group and 169 patients (conventional group) treated between January 2011 and the end of March 2016 were compared using propensity score matching. The frequency of laparoscopic resection was significantly larger in the synbiotic group (conventional group; 28% vs synbiotic group; 55%, *p*<0.001) and the amount of intraoperative bleeding was significantly smaller in the synbiotic group (median; conventional group, 700 ml vs synbiotic group, 200 ml; *p*<0.001). The postoperative SSI was significantly lower in the synbiotic group of six patients (5.2%) than in the conventional group of 30 patients (17.8%) (*p* = 0.002). Sixty patients in each group remained after propensity score matching. There was no significant difference in the incidence of SSI between the groups (conventional group, 15% vs synbiotic group, 6.7%; *p* = 0.239). In conclusion, preoperative synbiotic treatment did not reduce SSIs after hepatic resection.

## Introduction

Hepatic resection is one of the most challenging operations in general surgery. Advances in surgical techniques and perioperative care reduce postoperative complications, which are a major concern for surgeons.

Many patients recover without experiencing postoperative complications. However, some patients experience various types of infections, such as surgical-site infection (SSI), urinary tract infection, pneumonia and sepsis. Antibiotics are generally prescribed following surgery to prevent infections. However, the use of prophylactic antibiotics disrupts the gut microbiota, resulting in compromised barrier function of the intestinal epithelium.

Many functions of the immune system are controlled by the digestive system. Bacterial translocation, which is the invasion of bacteria from the gastrointestinal tract to other areas of the body, occurs through the loss of barrier integrity of the intestinal epithelium; age and disease are two factors that contribute to bacterial translocation.^(1,2)^

Recently, regulation of the intestinal flora has been studied, with the aim of improving barrier function. Administration of live microorganisms, called probiotics, is one method of modulating the intestinal flora. *Lactobacillus* and *Bifidobacterium* species are good probiotics.^(3)^ Prebiotics are non-digestible components, such as oligosaccharides or dietary fibers, that are fermented in the gut and efficiently increase the number of probiotic bacteria.^(4)^ The combinatorial administration of probiotics and prebiotics is called synbiotic treatment.^(4)^

Previous reports indicate that synbiotic treatment decreased the occurrence of complications after hepatic resection.^(5–7)^ These reports demonstrated this through the combined use of *Bifidobacterium* and oligosaccharides.

Therefore, we used a new combination of synbiotic treatment, which included *Clostridium butyricum* as a probiotic and dietary fiber as a prebiotic. *Clostridium butyricum* is a spore-forming, anaerobic, Gram-positive bacillus that produces the short-chain fatty acid (SCFA) butyrate. It was separated from the intestinal tract of a pig by Prazmowski and was named accordingly because butyric acid is the main metabolite it produces.^(8)^ The present study used MIYAIRI 588, which is a common strain of *Clostridium butyricum*, which stimulates polyclonal mucosal immune activity and inhibits multiplication of *Vibrio cholerae*, *Aeromonas hydrophila*, and *Shigella flexneri*.^(9,10)^ It also has preventive and therapeutic effects on enterohemorrhagic *Escherichia coli* (O157) infections,^(11)^ decreases the toxicity of *Clostridium difficile*,^(12)^ and suppresses progression of nonalcoholic steatohepatitis.^(13,14)^ MIYAIRI 588 is effective in treating antibiotic-associated diarrhea in children because it normalizes intestinal flora.^(15)^

Guar gum was used as a prebiotic and is a water-soluble natural polysaccharide made from the endosperm of the Guar bean plant. It improves hyperglycemia after meals,^(16)^ while decreasing total cholesterol and low-density lipoprotein cholesterol.^(17)^ In addition, diarrhea of malnourished children was significantly improved;^(18)^ symptoms of irritable bowel syndrome (IBS) patients significantly improved through administration of water-soluble guar gum in randomized controlled trials.^(19)^

The usefulness of water-soluble dietary fiber guar gum has also been reported in basic research. Administration of guar gum significantly reduced inflammation of the intestinal mucosa of a mouse model with colitis, promoted the growth of lactic acid bacteria strains, *in vitro*, and promoted stimulation of *Bacteroides* and *Parabacteroides*, which are beneficial for IBS and ulcerative colitis.^(20–22)^

In this study, we investigated if our synbiotic treatment using *Clostridium butyricum* and water-soluble guar gum would decrease infectious complications after hepatic resection.

## Materials and Methods

### Patients

There were 189 patients who underwent hepatic resection between January 2011 and March 2016. Among them, 169 patients were included as the historical control, or conventional, group. Thirteen patients with biliary tract reconstruction, 12 with combined resection of other organs, and five who had previously received other synbiotic treatments were excluded.

**Fig. 1:**
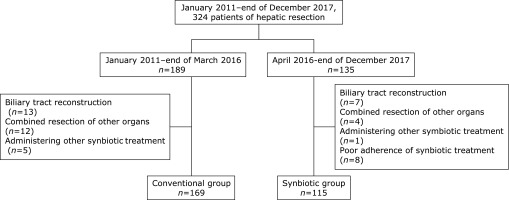
Study design and treatment groups.

Preoperative synbiotic treatment was prospectively started in April 2016. Among 135 patients who underwent hepatic resection between April 2016 and December 2017, 115 patients were included in the synbiotic group. Seven patients with biliary tract reconstruction, four with combined resection of other organs, one patient who received other synbiotic treatments, and eight patients with poor adherence to the synbiotic treatment were excluded (Fig. 1).

### Study protocol

*Clostridium butyricum* (MIYAIRI 588; Miyarisan Pharmaceutical Co., Ltd., Tokyo, Japan) was administered to the synbiotic group in a dose of 6.0 g/day, while 12.0 g/day of prebiotic was administered (Partially Hydrolyzed Guar Gum; Taiyo Kagaku Co., Ltd., Tokyo, Japan); both were administered preoperatively. The administration period lasted from one month preoperatively until the day before operation. (Clinical Research Registration; approval No. 28-103)

Synbiotics were administered for 2 weeks preoperatively and 11–14 days after surgery in previous reports on hepatectomy.^(6,7)^ Since the total period of administration of synbiotics was about one month in those studies, we decided to administer synbiotics for one month in our study. Furthermore, since IBS symptoms were improved by administering water-soluble guar gum for 12 weeks,^(19)^ we supposed that administration for one month was appropriate.

Preoperative background factors, blood test results, operative factors, postoperative complications, and hospitalization time were compared between the patients of the conventional group and the synbiotic group. The changes in white blood count (WBC), C-reactive protein (CRP) level and procalcitonin (PCT) level, which are postoperative inflammatory markers, were evaluated until postoperative day (POD) 3.

The *Clostridium difficile* (CD) antigen test was performed using patients in the synbiotic group whose fecal samples could be checked before and after synbiotic treatment. Fecal culture tests were performed to examine whether the CD toxin was produced for patients testing positive according to the CD antigen test positive (A or B positive).

A 1 g dose of cefazolin sodium was administered by intravenous drip infusion 30 min before the operation. The antibiotics were administered every 3 h during the operation, but no postoperative prophylactic antibiotics were administered.

Postoperative complications were defined as grade II or higher according to the Clavien-Dindo classification.^(23,24)^ Post-hepatectomy liver failure (PHLF) was defined according to the definition of the International Study Group of Liver Surgery (ISGLS).^(25)^ SSI was defined according to guidelines of the Center of Disease Control (CDC).^(26)^

### Ethics approval and consent to participate

This study conformed to the Clinical Research Guidelines and was approved by the ethical committee of Shiga University of Medical Science (approval No. 28–103). Informed consent was obtained from all patients or members of their families prior to surgery.

### Statistical analyses

Age, body mass index (BMI), and tumor size were expressed as mean ± SD and were compared using the Student *t* test. Other laboratory measures were expressed as medians with interquartile ranges and were compared using the Mann–Whitney *U* test. Differences in the values of categorical variables were compared using the chi-squared test or Fisher’s exact test.

To reduce potential bias on patient background and potential confounding variables in this observation study, propensity score matching was performed using nearest-neighbor matching without replacement. The factors selected for matching were single tumor vs multiple tumor, partial hepatectomy vs anatomical hepatectomy, initial hepatectomy vs repeat hepatectomy, laparoscopic hepatectomy vs open hepatectomy, amount of bleeding, and operation time, which had a postoperative effect. The scores were matched using a caliper width 1/5 logit of the SD.

A *p* value of <0.05 was considered statistically significant. All statistical analyses were performed using the R statistical package ver. 3.4.4 (The R Project for Statistical Computing, Vienna, Austria; https://www.r-project.org).

## Results

Table 1 displays patient background measures, blood test and operative factors before and after propensity score matching.

Before matching, the average age was 66 years in the conventional group and 68 years in the synbiotic group (*p* = 0.173). The number of females was significantly higher in the synbiotic group [conventional group, 34 females (20%) vs synbiotic group, 38 females (33%); *p* = 0.018]. The rate of medication of the proton pump inhibitor was similar between the groups [conventional group, 20 (11.8%) vs synbiotic group, 18 (15.6%); *p* = 0.378]. The conventional group included 80 patients (47.3%) with hepatocellular carcinoma (HCC), eight patients (4.7%) with intrahepatic cholangiocarcinoma (ICC), 74 patients (43.8%) with liver metastasis, and seven patients (4.1%) with other conditions. In contrast, the synbiotic group included 60 patients (52.2%) with HCC, one patient (0.9%) with ICC, 39 patients (33.9%) with liver metastasis, and 15 patients (13.0%) with other conditions. There was a statistically significant difference for all conditions between the groups (*p* = 0.006). However, etiology was similar between the groups [conventional group: hepatitis B virus (HBV) (3%), hepatitis C virus (HCV) (23%), negative of HBV and HCV (NBNC) (74%) vs synbiotic group: HBV (3.5%), HCV (23.5%), NBNC (73%); *p*>0.999].

The hepatic function reserve factors of albumin, aspartate aminotransferase (AST), alanine aminotransferase (ALT), bilirubin, platelet count, prothrombin activity, and indocyanine green retention rate at 15 min (ICGR15) were not significantly different between the groups.

The mean maximal tumor size was significantly larger in the conventional vs synbiotic group [3.5 cm vs 2.8 cm, respectively (*p* = 0.007)]. There were no significant differences between the prevalence of single or multiple tumors, and partial resection or anatomical resection. However, significantly more laparoscopic hepatic resections were seen in the synbiotic vs the conventional group [conventional group: 47 patients (28%) vs synbiotic group: 63 patients (55%); *p*<0.001]. The median operation time was significantly longer in the conventional group than the synbiotic group; 396 min vs 240 min, respectively (*p*<0.001). The median amount of blood loss was significantly smaller in the synbiotic group (conventional group: 700 ml vs synbiotic group: 200 ml; *p*<0.001).

The results after propensity score matching are described below. There was no significant difference in age, sex, prevalence of disease, etiology, and blood test findings after matching. The maximum tumor size, single or multiple tumors, partial or anatomical resection, initial or repeat resection, and laparoscopic or open resection were not different between groups.

Table 2 shows a comparison of the short-term results before and after propensity score matching. There were no significant differences among infectious complications in 40 patients (23.7%) of the conventional group and 19 patients (16.5%) of the synbiotic group before matching (*p* = 0.18). However, SSI was significantly lower in six patients (5.2%) of the synbiotic group than 30 patients (17.8%) of the conventional group (*p* = 0.002). Although other complications and the rate of bile leak were similar between groups, the PHLF was significantly higher in the conventional group [conventional group: grade A, 13 patients (7.7%); grade B, 16 patients (9.5%); grade C, 4 patients (2.4%) vs synbiotic group: grade A, 8 patients (7%); grade B, 1 patient (0.9%); grade C, 1 patient (0.9%); *p* = 0.007]. Hospitalization time was also significantly longer in the conventional group (conventional group, 12 days vs synbiotic group, 11 days; *p* = 0.001).

Results after propensity score matching demonstrated no significant differences between groups regarding all infectious complications, SSI, rate of bile leak, PHLF, or length of hospital stay.

**Fig. 2:**
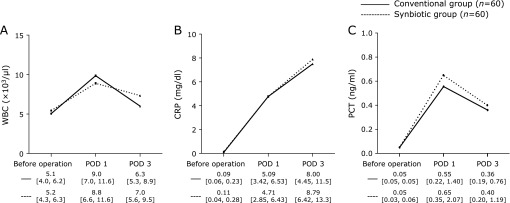
Graphical representations of (A) WBC, (B) CRP, and (C) PCT levels before surgery, and on POD 1 and 3. WBC, white blood count; CRP, C-reactive protein; PCT, procalcitonin; POD, postoperative day.

Inflammation was examined by WBC, CRP level, and PCT level before operation, and on POD 1 and 3. The median WBC was 5.15 × 10^3^ cells/ml before operation, 9.05 × 10^3^ cells/ml on POD 1, and 6.30 × 10^3^ cells/ml on POD 3 for the conventional group. In contrast, the WBC was 5.20 × 10^3^ cells/ml before operation, 8.80 × 10^3^ cells/ml on POD 1, and 7.00 × 10^3^ cells/ml on POD 3 for the synbiotic group. There were no significant differences between groups before operation, or on POD 1 and 3. Neither CRP level [conventional group: 0.09 mg/dl (before operation), 5.09 mg/dl (POD 1), 8.00 mg/dl (POD 3) vs synbiotic group 0.11 mg/dl (before operation), 4.71 mg/dl (POD 1), 8.79 mg/dl (POD 3)] nor PCT level exhibited significant differences between the groups before operation, or on PODs 1 and 3 (Fig. 2).

**Fig. 3:**
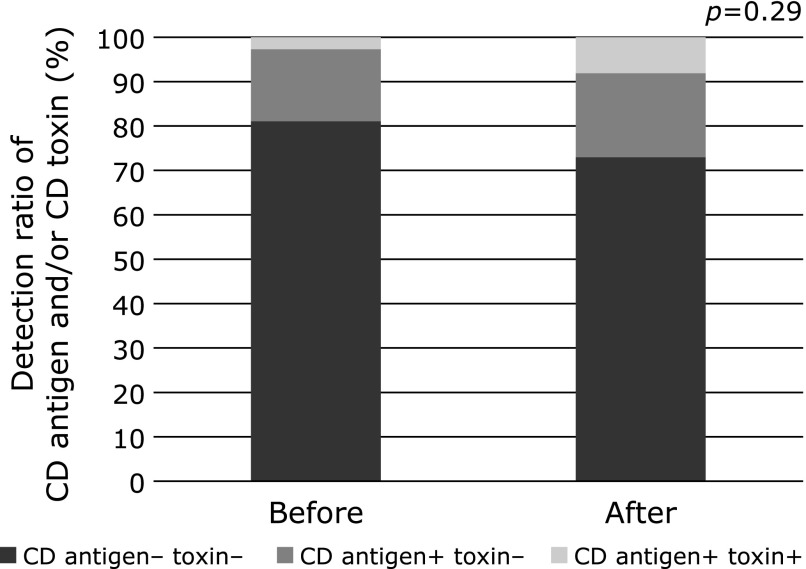
CD antigen and toxin test before and after synbiotic treatment. CD, *Clostridium difficile*.

Among the synbiotic group, fecal samples were examined in 36 cases before and one month after treatment. Seven patients (19.4%) were positive for CD antigen in the fecal examination before administration of synbiotic treatment. One patient among these seven (2.7%) produced the CD toxin. Ten patients (27.7%) were positive for the CD antigen one month after administration, three of which (8.3%) produced CD toxins (Fig. 3).

The changes in patient background parameters between before and after synbiotic treatment are shown in Table 3. The albumin, ALT, AST, total bilirubin, platelet count, prothrombin activity, estimate glomerular filtration rate, WBC, and CRP were measured before and after synbiotic treatment. These parameters had were not significantly different between the groups.

## Discussion

The results of the present study indicate that synbiotic treatment using a butyrate-producing bacterium and dietary fiber did not decrease the incidence of postoperative infection. Additionally, there were no significant differences in the amount of inflammation examined using WBC, CRP and PCT until POD 3 between the conventional and synbiotic groups. Furthermore, no significant decrease was found in the number of patients who were CD antigen positive vs those who were CD toxin positive.

Several studies have focused on perioperative synbiotic treatment and the effects of such treatments on prognosis. Some of these studies reported decreased postoperative infectious complications.^(6,7)^ In one study, it was found that perioperative synbiotic treatment increased preoperative natural killer T cell activity and lymphocyte count in patients with combined liver and extrahepatic bile duct resection with hepaticojejunostomy. As a result, it was reported that postoperative serum levels of interleukin-6 (IL-6), WBC and CRP decreased, while SCFA concentration in the feces increased.^(6)^ Perioperative synbiotic treatment also improved serum diamine oxidase activity, while decreasing serum IL-6 and CRP levels in patients with hepatic resection, regardless of the presence of liver cirrhosis.^(7)^ In patients with liver transplantation, synbiotic treatment also decreased postoperative infectious complications.^(27,28)^ Furthermore, in chronic pancreatitis patients, synbiotic treatment significantly decreased the incidence of postoperative sepsis, hospitalization time, and the duration of time in which postoperative antibiotics were administered.^(29)^

The usefulness of synbiotic treatment has been demonstrated; however, some results are still controversial. In regard to abdominal surgery, a study indicated that synbiotic treatment did not improve CRP and serum IL-6 levels or postoperative complications.^(30)^ However, synbiotic treatment increased the total organic acid and SCFA concentrations in feces, and decreased the incidence rate of infectious complications in elderly patients.^(31)^ In addition, it increased the diversity of the microbiome and decreased bacterial translocation following colorectal operations. However, these factors were not associated with a decrease in inflammation or postoperative complications in the study.^(32)^ Preoperative use of synbiotic treatment did not affect postoperative infections, even in patients receiving pancreatoduodenectomy.^(33)^ Therefore, the effects of preoperative synbiotic treatments cannot be generalized and further studies are necessary to elucidate the role of synbiotic treatments on the rate of infection.^(34,35)^

Recent meta-analyses demonstrated that preoperative synbiotic treatment decreased the rate of postoperative infections in general surgery.^(36–39)^ Over 30 papers included in these meta-analyses used *Lactobacillus* and/or *Bifidobacterium* as probiotics. In addition, most papers used oligosaccharides as prebiotics, and few used dietary fiber. Administration of probiotics or synbiotics reduced the occurrence of postoperative infections after colorectal surgery, hepatobiliary pancreatic surgery, and liver transplantation. Among them, these treatments were most effective for hepatobiliary pancreatic surgery. In most papers, synbiotics were administered both preoperatively and postoperatively, but the duration of administration varied in each paper. Based on these results, synbiotics administered both pre- and post-operatively using *Lactobacillus*, *Bifidobacterium*, and oligosaccharides likely reduce postoperative infectious complications. However, it is still unclear how long synbiotics should be administered.

Previous studies have focused on the perioperative use of synbiotic treatments comprised of *Bifidobacterium* species and oligosaccharides; however, it is unknown if this combination is the best for reducing SSI after hepatic resection. To the best of our knowledge, there are no other studies that utilize *Clostridium butyricum* and guar gum as synbiotic treatment.

We found that a symbiotic treatment using *Clostridium butyricum* and guar gum was not effective in preventing postoperative infectious complications. The biggest difference from previous papers was the type of selected drugs for synbiotics. Another difference was that symbiotic treatment was only administered preoperatively in the present study. Therefore, changing the combination of drugs and changing to preoperative and postoperative administration are future challenges.

This study has some limitations. The sample size was small, and the patients were from a single institution and not randomized. Therefore, large, randomized control studies including patients from multiple studies are necessary in the future.

In conclusion, synbiotic treatment using the butyrate-producing bacterium *Clostridium butyricum* and water-soluble guar gum fiber did not decrease postoperative infection or complications.

## Author Contributions

HI designed the research and analyzed the patient data. HI, MS, HM, HM, DY, KT, MK, SB, and MT performed the interventions. All authors read and approved the final manuscript.

## Figures and Tables

**Table 1:** Patients’ background factors between conventional group and synbiotic group before and after propensity score matching

		Before propensity score matching				After propensity score matching		
		Conventional group (*n* = 169)	Synbiotic group (*n* = 115)	*p* value		Conventional group (*n* = 60)	Synbiotic group (*n* = 60)	*p* value
Age		66.2 ± 12.6	68.2 ± 11.6	0.173		65.75 (13.67)	66.92 (13.61)	0.64
Gender	Female	34 (20.1)	38 (33.0)	0.018		10 (16.7)	19 (31.7)	0.087
	Male	135 (79.9)	77 (67.0)			50 (83.3)	41 (68.3)	
BMI (kg/m^2^)		22.70 (3.35)	22.98 (3.88)	0.524		22.17 (2.98)	22.65 (4.18)	0.466
Diabetes mellitus		57 (33.7)	29 (25.2)	0.148		21 (35.0)	16 (26.7)	0.429
Medication of PPI		20 (11.8)	18 (15.6)	0.378		7 (11.6)	9 (15.0)	0.789
Etiology	HBV	5 (3.0)	4 (3.5)	>0.999		3 (5.0)	1 (1.7)	0.317
	HCV	39 (23.1)	27 (23.5)			12 (20.0)	18 (30.0)	
	NBNC	125 (74.0)	84 (73.0)			45 (75.0)	41 (68.3)	
Alcohol abuse		47 (27.8)	32 (27.8)	>0.999		19 (31.7)	18 (30.0)	>0.999
Disease	HCC	80 (47.3)	60 (52.2)	0.006		31 (51.7)	29 (48.3)	0.06
	ICC	8 (4.7)	1 (0.9)			3 (5.0)	1 (1.7)	
	Meta	74 (43.8)	39 (33.9)			23 (38.3)	18 (30.0)	
	Other	7 (4.1)	15 (13.0)			3 (5.0)	12 (20.0)	
Albumin (g/dl)		3.8 [3.5, 4.1]	3.9 [3.5, 4.2]	0.306		3.9 [3.5, 4.2]	3.8 [3.5, 4.1]	0.459
ALT (IU/L)		23 [15, 36]	20 [13, 34]	0.145		20 [13, 33]	20 [13, 31]	0.836
AST (IU/L)		27 [21, 43]	26 [20, 34]	0.145		24 [18, 37]	26 [19, 33]	0.721
Bilirubin (mg/dl)		0.6 [0.5, 0.8]	0.6 [0.4, 0.8]	0.166		0.6 [0.5, 0.8]	0.5 [0.4, 0.7]	0.051
Platelet count (×10^3^/µl)		171 [130, 213]	177 [127, 221]	0.733		166 [129, 203]	178 [136, 220]	0.282
Prothrombin activity (%)		92 [84, 100]	96 [85, 103]	0.142		92 [85, 101]	93 [84, 100]	0.994
eGFR		70 [58, 85]	70 [58, 82]	0.788		72 [60, 86]	69 [59, 81]	0.258
ICGR 15 (%)		11.1 [6.2, 16.3]	8.7 [3.8, 15.9]	0.052		9.5 [5.0, 14.2]	8.1 [2.6, 13.2]	0.194
WBC (×10^3^/µl)		5.1 [4.1, 6.2]	5.1 [4.2, 6.2]	0.741		5.1 [4.0, 6.2]	5.2 [4.3, 6.3]	0.587
CRP (mg/dl)		0.14 [0.06, 0.36]	0.13 [0.05, 0.37]	0.777		0.09 [0.06, 0.23]	0.11 [0.04, 0.28]	0.723
PCT (ng/ml)		0.06 [0.04, 0.10]	0.05 [0.04, 0.06]	0.257		0.05 [0.05, 0.05]	0.05 [0.03, 0.06]	0.085
Tumor size (cm)		3.5 ± 2.4	2.8 ± 1.8	0.007		3.4 ± 2.3	3.0 ± 1.9	0.367
Tumor number (%)	Single	109 (64.5)	69 (60.0)	0.456		43 (71.7)	40 (66.7)	0.693
	Multiple	60 (35.5)	46 (40.0)			17 (28.3)	20 (33.3)	
Method (%)	Partial	95 (56.2)	72 (62.6)	0.326		34 (56.7)	35 (58.3)	>0.999
	Anatomical	74 (43.8)	43 (37.4)			26 (43.3)	25 (41.7)	
Repeat resection (%)		39 (23.1)	41 (35.7)	0.023		12 (20.0)	14 (23.3)	0.825
Laparoscopic resection (%)		47 (27.8)	63 (54.8)	<0.001		26 (43.3)	27 (45.0)	>0.999
Operation time (min)		396 [340, 503]	240 [185, 320]	<0.001		317 [240, 364]	284 [223, 378]	0.519
Blood loss (ml)		700 [305, 1,381]	200 [57, 527]	<0.001		305 [147, 703]	246 [75, 630]	0.385

BMI, body mass index; PPI, proton pump inhibitor; HBV, positive of hepatitis B antigen; HCV, positive of hepatitis C antibody; NBNC, negative of hepatitis B antigen and hepatitis C antibody; HCC, hepatocellular carcinoma; ICC, intrahepatic cholangiocellular carcinoma, Meta, metastatic liver tumor; ALT, alanine aminotransferase; AST, aspartate aminotransferase; eGFR, estimate glomerular filtration rate; ICGR 15, indocyanine green retention rate at 15 min; WBC, white blood count; CRP, C-reactive protein; PCT, procalcitonin. Data are expressed as median [interquartile range] and number (percent) without age, BMI and tumor size expressed as mean ± SD.

**Table 2:** Short-time results between conventional group and synbiotic group before and after propensity score matching

		Before propensity score matching				After propensity score matching		
		Conventional group (*n* = 169)	Synbiotic group (*n* = 115)	*p* value		Conventional group (*n* = 60)	Synbiotic group (*n* = 60)	*p* value
Infectious complications (%)		40 (23.7)	19 (16.5)	0.18		11 (18.3)	9 (15.0)	0.807
SSI (%)		30 (17.8)	6 (5.2)	0.002		9 (15.0)	4 (6.7)	0.239
	Deep	2 (1.2)	1 (0.9)	0.001		0 (0)	0 (0)	0.08
	Organ	13 (7.7)	5 (4.3)			4 (6.7)	4 (6.7)	
	Surface	15 (8.9)	0 (0.0)			5 (8.3)	0 (0.0)	
Other complications (%)		39 (23.1)	24 (20.9)	0.771		8 (13.3)	13 (21.7)	0.337
Bile leakage (%)		14 (8.3)	11 (9.6)	0.832		4 (6.7)	5 (8.3)	>0.999
PHLF (%)	A	13 (7.7)	8 (7.0)	0.007		4 (6.7)	5 (8.3)	0.367
	B	16 (9.5)	1 (0.9)			3 (5.0)	0 (0.0)	
	C	4 (2.4)	1 (0.9)			0 (0.0)	1 (1.7)	
Hospital days		12 [9, 18]	11 [9, 14]	0.006		10 [8, 13]	10 [9, 15]	0.495

SSI, surgical site infection; PHLF, post-hepatectomy liver failure. Data are expressed as median [interquartile range] and number (percent).

**Table 3:** Changes of the patient’s background parameters in before and after synbiotic treatment

	Before synbiotic treatment (*n* = 115)	After synbiotic treatment (*n* = 115)	*p* value
Albumin (g/dl)	3.9 [3.4, 4.1]	3.9 [3.5, 4.2]	0.546
ALT (IU/L)	23 [16, 31]	20 [13, 34]	0.113
AST (IU/L)	27 [22, 43]	26 [20, 34]	0.114
Bilirubin (mg/dl)	0.7 [0.5, 0.8]	0.6 [0.4, 0.8]	0.317
Platelet count (×10^3^/µl)	185 [134, 231]	177 [127, 221]	0.402
Prothrombin activity (%)	96 [87, 109]	96 [85, 103]	0.09
eGFR	69 [58, 84]	70 [58, 82]	0.991
WBC (×10^3^/µl)	4.9 [4.0, 6.3]	5.1 [4.2, 6.2]	0.807
CRP (mg/dl)	0.11 [0.07, 0.30]	0.13 [0.05, 0.37]	0.763

ALT, alanine aminotransferase; AST, aspartate aminotransferase; eGFR, estimate glomerular filtration rate; WBC, white blood count; CRP, C-reactive protein. Data are expressed as median [interquartile range].

## Abbreviations

ALT: alanine aminotransferase
AST: aspartate aminotransferase
BMI: body mass index
CD: *Clostridium difficile*
CRP: C-reactive protein
HBV: hepatitis B virus
HCC: hepatocellular carcinoma
HCV: hepatitis C virus
ICC: intrahepatic cholangiocarcinoma
IBS: irritable bowel syndrome
NBNC: negative of HBV and HCV
PCT: procalcitonin
PHLF: post-hepatectomy liver failure
POD: postoperative day
SCFA: short-chain fatty acid
SSI: surgical-site infection
WBC: white blood count
